# ReCANVo: A database of real-world communicative and affective nonverbal vocalizations

**DOI:** 10.1038/s41597-023-02405-7

**Published:** 2023-08-05

**Authors:** Kristina T. Johnson, Jaya Narain, Thomas Quatieri, Pattie Maes, Rosalind W. Picard

**Affiliations:** 1 Massachusetts Institute of Technology, MIT Media Lab, Cambridge, MA USA; 2 Massachusetts Institute of Technology, Lincoln Laboratory, Lexington, MA USA

**Keywords:** Biomedical engineering, Biomarkers, Computational science, Communication, Autism spectrum disorders

## Abstract

Nonverbal vocalizations, such as sighs, grunts, and yells, are informative expressions within typical verbal speech. Likewise, individuals who produce 0–10 spoken words or word approximations (“minimally speaking” individuals) convey rich affective and communicative information through nonverbal vocalizations even without verbal speech. Yet, despite their rich content, little to no data exists on the vocal expressions of this population. Here, we present ReCANVo: **Re**al-World **C**ommunicative and **A**ffective **N**onverbal **Vo**calizations - a novel dataset of non-speech vocalizations labeled by function from minimally speaking individuals. The ReCANVo database contains over 7000 vocalizations spanning communicative and affective functions from eight minimally speaking individuals, along with communication profiles for each participant. Vocalizations were recorded in real-world settings and labeled in real-time by a close family member who knew the communicator well and had access to contextual information while labeling. ReCANVo is a novel database of nonverbal vocalizations from minimally speaking individuals, the largest available dataset of nonverbal vocalizations, and one of the only affective speech datasets collected amidst daily life across contexts.

## Background & Summary

Nonverbal vocalizations, such as grunts, yells, and squeals, are an important part of communication^1^. Traditionally, human-based studies of affect and communication using nonverbal vocalizations have focused on pre-verbal vocalizations in infants^2,3^ or on nonverbal vocalizations that occur amidst typical word-based speech like moans and sighs^4,5^. Yet, for non- and minimally speaking individuals who produce zero or only a handful of spoken words (denoted here as mv* individuals), nonverbal vocalizations convey important communicative and affective information. Note that we use the term mv* (“M-V-star”) to refer to a sub-population of non- and minimally speaking individuals. These individuals have limited expressive language through verbal speech, alternative and augmentative communication (AAC) devices, and signed languages, though they use vocalizations and other nonverbal expressions such as gestures, facial expressions, and vocalizations as effective modes of communication. To our knowledge, nonverbal vocalizations as communication from mv* individuals have not been systematically studied, due in part to a lack of access to data from this community. Here, we present the first dataset of nonverbal vocalizations from mv* individuals labeled for affect and communicative function. The goal of this dataset is to spur further investigation into the acquisition, analysis, and reciprocation of non-speech vocalizations from minimally speaking individuals.

The study of nonverbal vocalizations with mv* individuals presents unique challenges. The population is relatively small, comprising approximately 1–2 million in the United States^6–8^, and they are geographically distributed^9,10^. The resource burden on this population is high^11,12^, so studies must be designed thoughtfully to minimize the time, effort, and inconvenience of participation. Additionally, this population is highly heterogeneous, including diagnoses of autism spectrum disorder (ASD), genetic neurodevelopmental disorders, cerebral palsy (CP), and other global developmental delays, and the specific etiologies of certain behaviors and symptoms are often not known^13–15^. For example, a person may not speak due to motor planning difficulties, cognitive delays, differences in social motivation, some combination thereof, or alternative causes. In addition, the abilities and/or behaviors affecting communication can evolve over time, further augmenting the heterogeneity of this group and motivating the need for quantitative longitudinal data from each individual.

Furthermore, an individual may also communicate in one way in their home or family but completely differently or not at all in a laboratory setting or with examiners^16,17^, underscoring the need for *in-situ* environmental contexts, familiar people, and real-world data collection. The sparsity and diversity of vocalizations requires a longitudinal approach to collect a representative sample of vocalizations from each person and necessitates a data processing methodology that accounts for the spontaneity of the vocalizations and the noisy, variable audio environment of real life. Finally, understanding these vocalizations requires familiarity and camaraderie with the communicator. Since most mv* communicators cannot directly provide word-based labels, labels from a person with a long-term relationship with the communicator are the closest obtainable ground truth. Moreover, labels denoted in-the-moment have access to the full multimodal context of the communication exchange, such as body language, gestures, and environment, increasing the fidelity of the labels.

Previously collected available datasets of nonverbal vocalizations have focused on vocalizations that occur amidst typical verbal speech using actors^18,19^ or recordings scraped from the web^5,20^. There is also a body of work analyzing infant vocalizations^21–25^, though few available datasets exist^5^. Likewise, affective speech datasets have predominantly been collected in lab environments with actors^26,27^. Naturalistic speech datasets have only been collected with typical verbal speech and are often only collected during specified activities^28–30^, limiting their ability to capture the breadth of affective expressions that occur across the varied experiences of daily life. To our knowledge, the ReCANVo dataset is the first dataset of affective speech vocalizations collected fully “in the wild,” across settings and activities.

This dataset presents over 7000 samples of labeled real-world vocalizations from eight mv* communicators. It is, to our knowledge, the only dataset of nonverbal vocalizations from non-speaking individuals, the largest available dataset of nonverbal vocalizations, and one of the only datasets collected in real-world settings with personalized labels with any population. In addition, basic demographic information and communication profiles are provided for each individual to offer additional insight into how nonverbal vocalizations are used by mv* communicators. Improved understanding of nonverbal vocalizations could contribute to the development of technology to augment communicative interactions^31^ and help answer critical questions around the emergence of language and communication across all stages of human development and expression. We hope that the published dataset will engage other researchers in this critical field of study.

## Methods

### Participants

Participants were recruited through conversations with community members and word of mouth for a larger study examining how mv* individuals use nonverbal vocalizations to communicate and how communication exchanges might be augmented by technology^32^. In the ReCANVo dataset presented here, we included participants who had collected data for at least ten recording sessions to ensure a sufficient number of captured vocalizations across a diversity of settings. These participants ranged in age from 6–23 years old and included diagnoses of autism spectrum disorder (ASD), cerebral palsy (CP), and genetic disorders. They all had fewer than 10 spoken words or word approximations, per parent report (see Table 1). The gender distribution of this sample (6 males, 2 females) reflects the gender distribution among the larger diagnostic categories (e.g., approximately 3.8 males are diagnosed with ASD for every 1 female^33^). No participants were excluded on the basis of age, diagnosis, or other measures in order to capture a broad cross section of this unique and understudied population of communicators.

**Table 1:** Participant demographics.

Participant ID	Gender	Age (year range)	Diagnoses affecting speech and/or language	Time span of included data (weeks)	Number of spoken words or word approximations (parent report)
P01	M	18–25	Autism, Down syndrome (DS)	64	0
P02	M	18–25	Autism	7	4
P03	M	6–9	Autism, Rare genetic disorder	16	0
P05	F	9–12	Autism	11	0
P06	M	9–12	Autism, Cerebral palsy (CP)	4	3
P08	F	6–9	Autism	20	0
P11	M	9–12	CP	19	1
P16	M	6–9	Autism	10	5–8

Importantly, the focus of this initial work and dataset release was on capturing deep, longitudinal, ecologically valid data from a range of participants. This process involved creating new real-world data acquisition methodologies and post-processing signal analysis techniques. Following best practices for novel research with specialized populations^34,35^, we utilized a highly iterative and participatory co-design process with a small number of participants^36^. Our dataset includes a variety of different recording settings over time spans of months, along with personalized labels for each participant. Given the limited prior work on real-world vocalizations from minimally speaking individuals, this depth-focused approach was a critical first step towards understanding the heterogeneity of this population, and we look forward to future work expanding our understanding of vocalizations from mv* communicators.

**Fig. 1:**
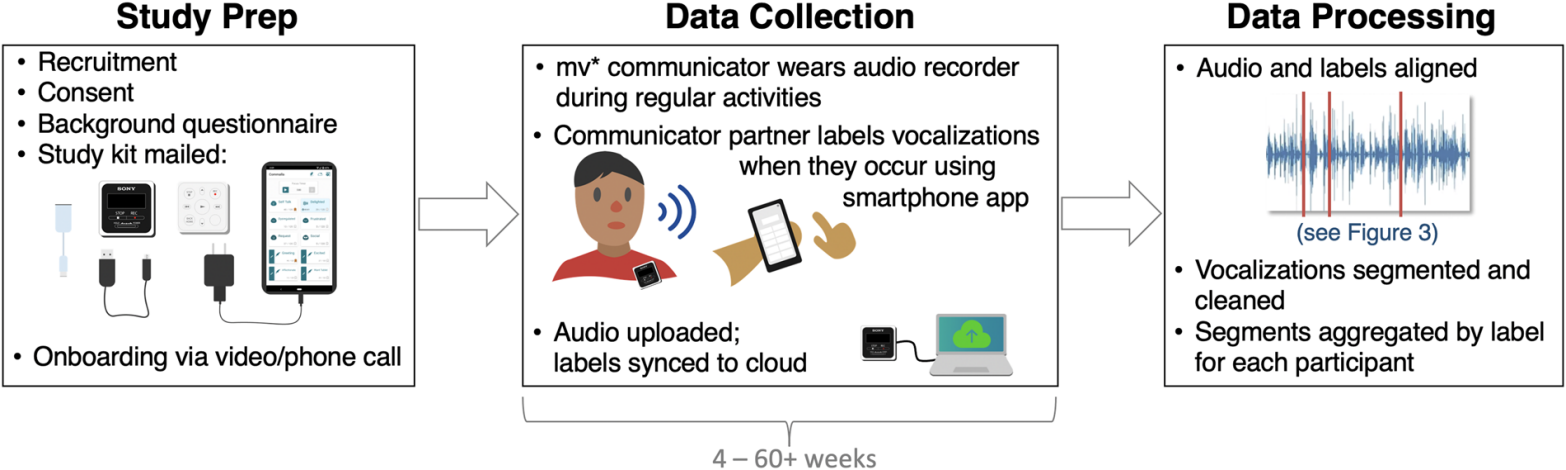
Schematic for the ReCANVo dataset creation.

Table 1 provides basic demographic information on the eight mv* communicators included in this dataset. Age ranges are provided to bolster anonymity. Figure 1 provides a schematic of the overall study setup and data processing. The study and data collection protocol was approved by the Committee on the Use of Humans as Experimental Subjects (COUHES), the institutional review board (IRB) at the Massachusetts Institute of Technology (MIT). Informed consent or assent was obtained from all participants. A parent or legal guardian provided consent for mv* participants, who were all considered minors for the purposes of consent, and special attention was given to the assent of the non-speaking communicators throughout the study. Families were given flexibility to record audio when it was most convenient for them and could terminate the recording session at any time. They were also given control over when and whether to share the audio data with the researchers and were specifically asked if they wanted to opt in to sharing the de-identified vocalization clips publicly, instead of an opt-out policy. All data clips were manually checked to ensure no identifying information (such as spoken names) remained.

### Terminology

In this paper, we use the term *communicator* to refer to the mv* individual who was producing the vocalizations of interest. We use the term *communication partner* or *labeler* to refer to the individual — often a parent or family member — who knew the communicator well and was providing the real-time labels of the vocalizations. The term *participant* refers to both communicators and communication partners as they worked jointly during data collection. Finally, we use the word *communication* to refer to the exchange of information between people. This definition includes, but is not limited to, non-speech vocalizations that convey information to a listener or communication partner. These sounds do not need to be intentional to be informative (similar to the way a shout or cry may convey affect or other information to listeners – whether the sound was produced intentionally or not, and whether it was directed to another person or not).

### Real-world audio data

Audio data were collected using a Sony IDC-TX800 wearable audio recorder in 16-bit, 44.1 kHz stereo (see Fig. 1). Magnets were attached to the back of the recorder so that it could be comfortably attached to the communicator’s clothing (see previous work for a detailed discussion of this methodology^36^). Some participants had tactile sensitivities that prevented even the lightweight recorder from being attached to the clothing and were instructed to place or hold the recorder near the communicator.

All data were collected and labeled remotely, in the participants’ homes and natural environments. This remote administration allowed us to reach a geographically distributed population and produced highly naturalistic data. Data collection kits were mailed to participating families. These kits included the Sony audio recorder, a mobile phone with a custom app for in-the-moment labeling, peripheral cables, and instructions (see Fig. 1). Participants were encouraged to go about their typical daily activities while recording and to label at their convenience to lower the burden of integrating data collection into daily life. This naturalistic data acquisition method resulted in intentionally sparsely labeled recordings.

### Real-time labeling app

**Fig. 2:**
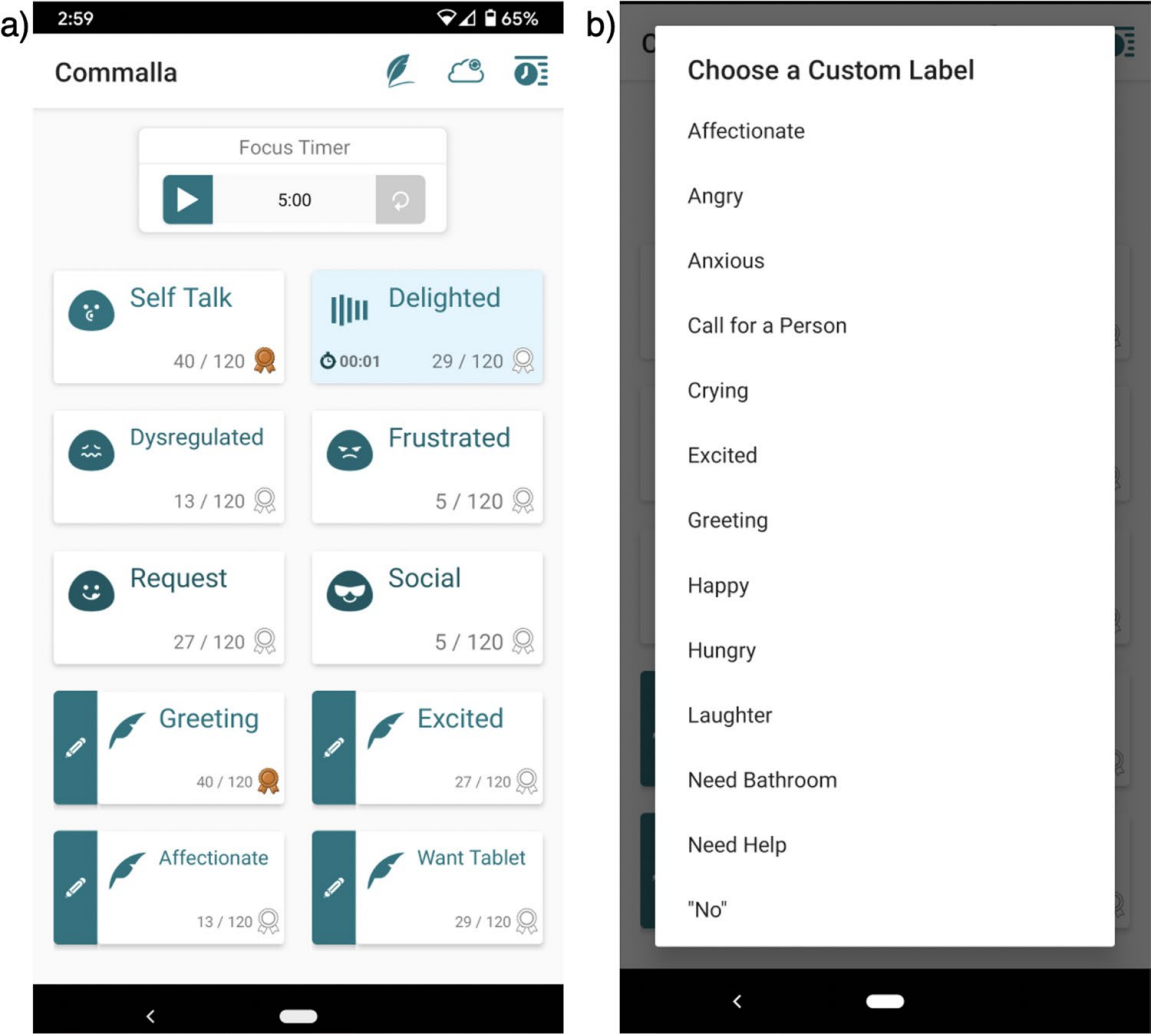
Custom labeling smartphone application provided to participants. (**a**) Main interface for in-the-moment labeling. Labels were tapped to indicate the start of a vocalization and tapped again to indicate the end of a vocalization. After a label was pressed, an animation appeared on the label (shown on the “Delighted” button) to remind the user which label was active. Labels and audio from a time-synchronized wearable recorder were aligned during post-processing. The six labels at the top of the screen were the same for all participants, while the four labels at the bottom of the screen could be customized for each participant. The “Focus Timer” was provided so participants could keep track of how long they had been labeling. (**b**) Partial list of preset options for the four customizable labels. See Table 1 for the complete list of labels used by participants in this dataset.

Vocalizations were labeled in real-time using a custom-built smartphone application (see Fig. 2). The app included 6 labels that were identical for all participants, as well as 4 labels that could be customized by each family from a list of 25 preset options (see Fig. 2b). These labels were selected for this study based on interviews with families of minimally speaking communicators and conversations with speech-language pathologists. They were designed to span a range of common affective states that might be associated with a sound (e.g., Frustration, Delight), as well communicative expressions that many mv* individuals conveyed via vocalizations (e.g., Request). While broad, the category of “social” vocalizations was included because it is important for unfamiliar communication partners to recognize and understand social calls from the communicator even if they were not able to precisely identify a more specific meaning. Descriptions of the 6 pre-determined labels were provided to labelers and are outlined here:

- **Frustration:** Vocalizations that are associated with being frustrated or angry. These vocalizations are typically made in response to a specific situation (e.g., not getting what is wanted).
- **Delight:** Vocalizations that are associated with being excited, very happy, or gleeful.
- **Dysregulation:** Vocalizations that are associated with being irritated, upset, agitated, bored, uncomfortable, understimulated, overstimulated, or generally distressed. These vocalizations may be made involuntarily or without a known communicative function; however, they convey a dysregulated affective state and are well understood by listeners who know the communicator well, making them deeply informative and important to capture.
- **Self-talk:** Vocalizations that are associated with being content, happy, or relaxed and often seem playful or exploratory in nature. These vocalizations generally appear to be made without an overt communicative function (i.e., the individual seems to be making the vocalizations to him/herself). For some individuals (of any age), these vocalizations may sound similar to canonical babbling, singing, or other vocal play heard in young typically-developing children.
- **Request:** Vocalizations that are associated with making a request.
- **Social:** Vocalizations that are social in nature and are not more accurately described by a different term or more specific social term (e.g., “greeting,” “call for a specific person”).

Label descriptions were shared with families. Particularly, the distinctions between “dysregulation” and “frustration” and between “self-talk” and “delight” were discussed in depth with families. For example, dysregulated vocalizations tend to be more general and less specific to a situation than other negative affective expressions like frustration (e.g., being frustrated that you cannot have a snack versus being dysregulated due to malaise, under/overstimulation, or some broader cause). Likewise, self-talk vocalizations differ from delight vocalizations in both function (e.g., delight vocalizations are more likely to be made in response to pleasurable circumstances to convey delight whereas self-talk vocalizations are generally made to one’s self and may have no specific prompt or obvious meaning) and arousal (“very happy or gleeful” versus “context, happy, or relaxed”). These states also often differ in contextual information and other nonverbal cues, such as facial expressions and body language.

During the intake screening, parents indicated the ways in which their mv* communicator consistently used vocalizations. Participants were instructed to use only those labels for which the communicator had a vocalization. Not every communicator produced vocalizations associated with every category of sound. For example, some communicators did not have “social” vocalizations and others did not produce “dysregulated” vocalizations. Thus, these labels were not used for those individuals.

The app also included the option for 4 semi-personalizable labels. By using a combination of both pre-determined labels that were consistent across all participants and labels that could be customized for each individual, we were able to capture a personalized representative sample of the types of vocalizations that these individuals produced. We were also able to capture vocalization functions that might be uncommon across all participants but very meaningful to that participant, such as “help”, “yes”, “request tablet”, or “hungry”. Not all participants used the preset options; they were only used if the family indicated that the communicator had additional specific vocalizations that they wanted to capture. Hence, the these additional labels were specific to each participant (see Table 2 for the labels used per participant). Participants were guided through selecting these semi-personalizable labels from a preset list of words during app setup, after which these labels were not changed. Thus, each participant had a fixed set of 6–10 labels to use throughout their study.

**Table 2:** The number of vocalizations included in the dataset, organized alphanumerically by participant and communicative function.

Vocalization Label	P01	P02	P03	P05	P06	P08	P11	P16
delighted	357	43	25	235	227	39	207	139
dysregulated	212	0	302	116	5	13	22	34
frustrated	150	56	47	283	30	781	27	162
request	130	13	61	6	124	44	22	19
self-talk	564	34	55	286	56	503	33	354
social	182	247	0	0	1	93	52	59
affectionate	0	126	0	0	3	0	0	0
bathroom	20	0	0	0	0	0	0	0
dysregulation-bathroom	18	0	0	0	0	0	0	0
dysregulation-sick	74	0	0	0	0	0	0	0
glee	1	0	7	0	0	0	0	0
greeting	0	0	0	0	0	0	3	0
happy	0	0	0	61	0	0	0	0
help	0	0	0	24	0	0	0	0
hunger	0	0	0	4	0	0	0	0
laughter	0	38	8	13	0	42	0	0
more	0	0	0	0	0	22	0	0
no	0	0	0	0	0	0	0	12
protest	0	0	20	0	0	1	0	0
tablet	0	0	0	0	0	7	0	0
yes	0	0	0	0	123	0	0	0

The first six vocalization labels were the same across all participants while the rest of the labels were optional semi-customizable labels chosen by each participant.

Note that vocalizations produced by mv* individuals might have multiple simultaneous meanings (i.e., a “frustrated request”) or ambiguous meanings. Labelers were asked to only label a vocalization if they had high confidence in their interpretation of the function of the sound and to assign the most appropriate label. As a result, there is one intended label per recorded vocalization.

### Vocalization labeling procedure

While the communicator was wearing the recorder, the communication partner labeled vocalizations as they were produced. For example, a communicator might request a drink by vocalizing and gesturing toward a cup. The communication partner would then tap the “Request” label on the smartphone labeling app.

Labelers were asked to achieve as close to a 1:1 mapping between a vocalization and a label as possible. However, not all participants followed this instruction closely; some participants designated long periods of time containing multiple vocalizations as a single label. These labeling techniques are further discussed in the preprocessing methods (e.g., Alignment of Audio and Labels) below. The app required labelers to indicate a ‘start’ and ‘end’ time for a vocalization by tapping the corresponding label. A color change and animation appeared on a label that had been ‘started’ to visually indicate which label was active (see Fig. 2a).

### Additional study details

At the beginning of the study, each participant had a personal meeting or call with the research team to review the study protocol, ask any questions, and set up the labeling app. Consent and/or assent was acquired from each participant. In addition, participants were provided with multimodal instructions to aid understanding and reliable data acquisition, including a series of video instructions (https://bit.ly/commalla-youtube) as well as a website with written and illustrated instructions. Step-by-step instructions were also included in each mailed data collection kit and provided as a PDF for each participant. Finally, participants were given the researcher’s contact information and encouraged to reach out with any questions as the study progressed.

The study was designed to be flexible and minimize the time and effort burden on participating families. Participants could choose the pace, location, and activities for data collection. While this flexibility resulted in some variability in the collected data between participants, it was critical in enabling this real-world first-of-its-kind data collection with a specialized population.

### Alignment of audio and labels

Participants uploaded recorded audio files via a cloud-based file sharing platform. Labels from the app were synced directly to a web server managed by the research team. The clock on the recorder and the app were synced to the same internet-accessible atomic clock (https://time.is) prior to shipping the equipment.

**Fig. 3:**
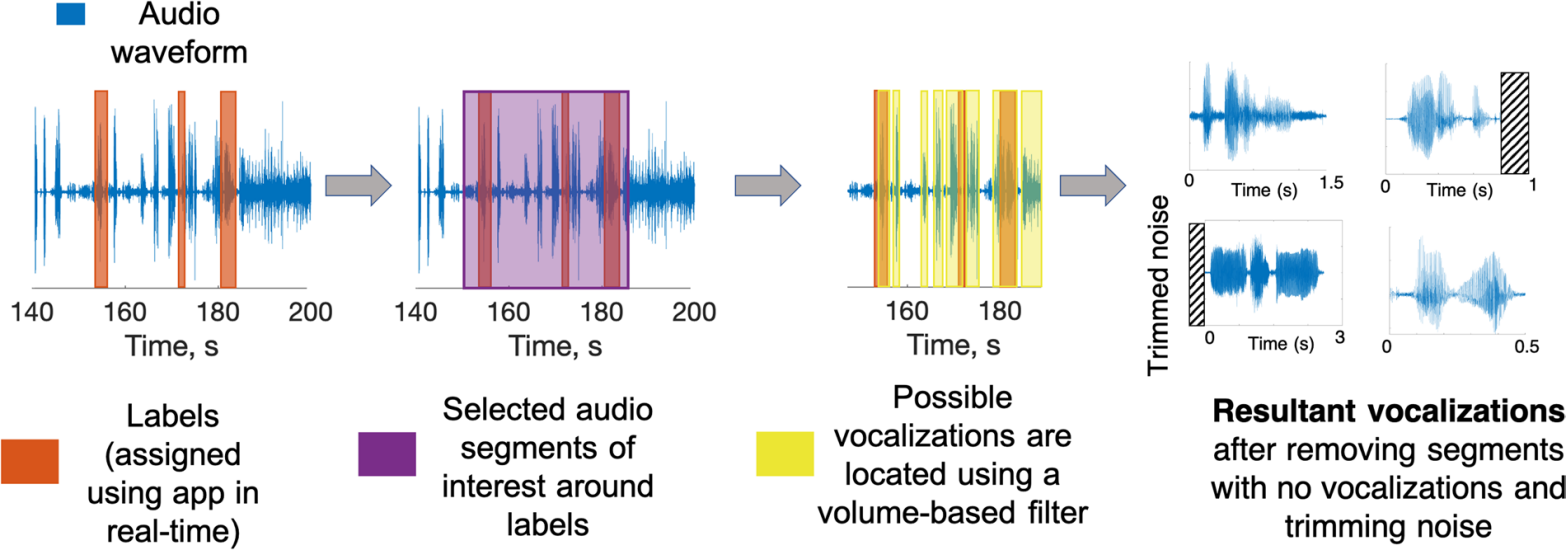
The audio data and real-time labels from the app were processed post-hoc to align labels with vocalizations. A volume-based filter was used to isolate audio segments of interest. Segments temporally near a label were assigned to that label. A researcher listened to each segment to ensure it contained a vocalization and, if necessary, trimmed excess noise around the vocalization.

The audio recordings and label information were then processed to isolate vocalizations of interest and align them with the assigned vocalization labels. Because participants were instructed to record and label at their discretion, we first isolated regions of audio that were temporally near labels (see Fig. 3, purple regions). Then, because the recorder was attached to the communicator’s clothing or placed nearby, a volume-based filter was used to isolate smaller audio segments within these regions that were likely to be vocalizations (see Fig. 3, yellow regions). The volume filter thresholds were selected for each session based on the recording levels during that session; they ranged between −20 and −45 dB. Vocalizations were considered distinct (separate vocalizations) if they were separated by approximately 250–450 ms of silence, determined heuristically based on the volume levels and background noise of that session’s recording. Additional information on alignment and segmentation is detailed in other work^37^.

**Fig. 4:**
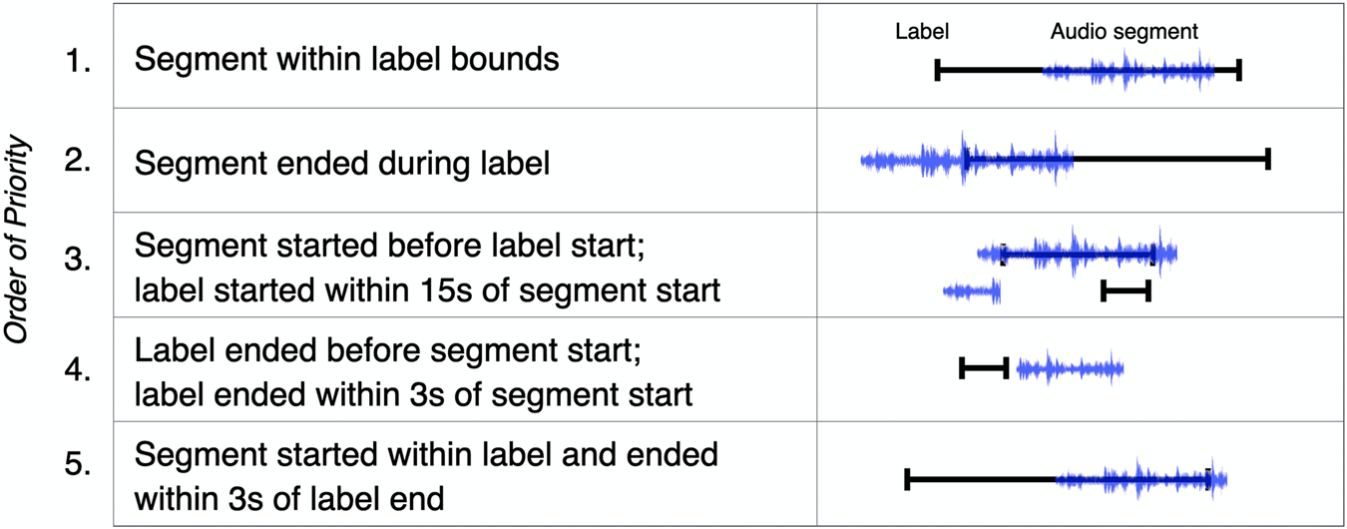
Illustration of rules for assigning labels to segments. The rule numbers in the figure correspond to the descriptions in the body of the paper.

Isolated segments were then assigned a label based on the following rules (see Fig. 4):

1. The audio segment was within the label bounds.
2. The audio segment ended during a label. This timing occurred naturally when a labeler pressed the label after hearing and recognizing a vocalization.
3. The audio segment started before the label started, and the label began within 15 s of the segment start. This threshold was determined after listening to hundreds of raw audio files. It accounts for the human labeling delay associated with in-the-moment labeling in real-world setting. In many cases, labels could be assigned to vocalizations even with this long delay due to the sparse nature of vocalizations from the mv* population (i.e., no other vocalizations were made during that time).
4. The label ended 3 seconds or less before the segment started. This alignment strategy was primarily necessary for series of vocalizations that occurred amidst multiple identical labels. For example, a communicator might produce four or five frustrated vocalizations sequentially, but owing to human delay and the realities of attending to the communicator’s needs during real-world data collection, only a few vocalizations might be labeled. However, the temporal proximity of the labels and the vocalizations still allowed for label assignment.
5. The segment started within a label and ended within 3 s of the label end. Because some labels encompassed multiple vocalizations, some segments began after the label had been pressed. This timing threshold is shorter to account for the possibility that a labeler may have ended a label because the vocalization type had changed and the current label was no longer accurate.

These rules were determined heuristically by comparing the timings of labels to the full-length audio files. The background audio and conversational exchange in the audio files provided context to determine if a label matched a given vocalization. If multiple distinct labels satisfied the rules above, a single label was selected, prioritizing the label with the rule with the lowest number in the list above. Note, by design, that not every vocalization in a recording was assigned a label since participants were instructed to label during free moments while recording. Unlabeled vocalizations were not included in this dataset. Provided labels were comprehensively included in the dataset. On average, each label corresponded to 2–3 final vocalization segments^37^.

After labels had been assigned to audio segments, a researcher listened to each labeled audio segment. Segments that did not contain vocalizations were discarded. Segments that contained additional noise or voices before or after a vocalization were manually trimmed. Vocalizations were defined as any clear sound from the communicator that could be associated with a label. This definition encompassed both voiced and non-voiced vocalizations, including word-like approximations with clear vowel-consonant sounds, as well as sounds like grunts, moans, yells, laughter, and breathy vocalizations. Every audio file included in the dataset has been manually confirmed to contain a vocalization. The ReCANVo dataset was intended to be representative of real-world data, so some trimmed vocalizations contain background sounds.

## Data Records

All dataset files described below, including raw data files, can be found on Zenodo^38^: 10.5281/zenodo.5786859.

The dataset contains audio recordings of segmented vocalizations, labeled by vocalization meaning or function. The vocalizations are 16-bit, 44.1 kHz .wav files that are organized by assigned label. A .csv file is provided that has the name of each vocalization file and the corresponding participant ID and vocalization label. In addition, communication profiles are provided for each participant in a separate .csv file. This background information was shared by each mv* communicator’s parent as part of a study intake questionnaire. The communication profile includes the communication modalities used by the participant (e.g., AAC use, gestures, vocalizations), the number of spoken word and word approximations produced by the communicator, and feedback on if and how the communicator uses vocal sounds across various communicative and affective categories.

The filenames of each audio recording have the following format:

$${\rm{YYMMDD}}\_{\rm{HHMM}}\_{\rm{SH}}\_{\rm{SM}}\_{\rm{SS}}{\rm{.ss}}\,-\,{\rm{EH}}\_{\rm{EM}}\_{\rm{ES}}{\rm{.ss}}$$

where YYMMDD_HHMM indicates the year (YY), month (MM), day (DD), hour (HH), and minute (MM) of an audio file, respectively. The start and end times of a vocalization *relative to the file start time* are given by SH, SM, SS, ss and EH, EM, ES, ss, indicating the vocalization start or end hour, minute, second, and sub-second, respectively. These times are included in the filename to provide additional information regarding the longitudinal nature of the dataset. Users of the dataset should note that these times are approximate and were determined using the segmentation and cleaning process described above. For P01 specifically, the start and end times of the vocalizations were estimated post-hoc using an autocorrelation and have known errors.

The ReCANVo dataset includes 7,077 vocalizations collected longitudinally with 8 mv* communicators. Table 2 shows the number of vocalizations in the dataset for each participant and vocalization type. To our knowledge, the ReCANVo dataset is the first dataset of nonverbal communication that occurs independent of typical verbal speech, the largest existing dataset of nonverbal vocalizations, and the first public dataset of affective speech collected longitudinally during day-to-day life across settings.

## Technical Validation

We identified three possible sources of labeling error:

1. Accidental labels (e.g., a labeler accidentally tapping the wrong label on the app)
2. Inaccurate vocalization-label alignment (e.g., labels being incorrectly matched with a vocalization audio during post-processing)
3. Inaccurate interpretation of a vocalization by a communication partner.

To mitigate the first two sources of error, a researcher listened to the audio surrounding each labeled vocalization. A researcher also listened to full-length audio recordings for each participant at least every two weeks of collected data. The surrounding context from the audio recording, such as spoken dialogue that confirmed an emotional label (E.g., “I know you want to go outside and we can’t. That’s frustrating.”) or answered a communicator’s request (E.g., “Is this the snack you wanted?”), was used to confirm that the assigned labels matched the audio context near the label. Because of the longitudinal nature of the study, some clock drift (~10 seconds or less) was observed for some participants. This drift was manually determined and accounted for when aligning the labels with vocalizations.

To mitigate the third source of error (i.e., incorrect interpretation by the communication partner), only communication partners who were deeply familiar with the mv* communicator and their communication style provided labels. In addition, partners were instructed to only label vocalizations that they felt like they could confidently interpret. However, any interpretation of a vocalization remains, at best, an interpretation. We hope that as additional knowledge and communication technology for mv* communicators becomes available, it will be possible to obtain ground truth meaning of these vocalizations directly from communicators.

In addition, there were expected sources of noise associated with real-world data, including environmental noise (e.g., wind, movement, background toys and electronics), overlapping voices, and intensity changes due to variable location of the recorder. Many extraneous sources of noise were removed during the segmentation process or through manual trimming; however, vocalization segments of all qualities were included here to ensure naturalistic, real-world data transfer.

## Data Availability

We used the Python programming language for the data processing described above. Volume segmentation was implemented using the *pydub* libary. The label assignment algorithm is summarized in Fig. 3. The code is available as part of our dataset in Zenodo: 10.5281/zenodo.5786859.
